# Supplementary material for: Assessing the Impact of the Methane Inhibitors 3-Nitrooxypropanol (3-NOP) and Canola Oil on the Rumen Anaerobic Fungi
Source: Animals (Basel). 2025 Apr 27;15(9):1230. doi: 10.3390/ani15091230 (PMC12071074; doi:10.3390/ani15091230)
Supplement: Supplementary file 1 [file animals-15-01230-s001.zip › ohara_et_al_2025_inhibitor_fungi_SupplementalTables.pdf]

Supplementary Tables

Table S1: Summary of Shannon diversity index values for each treatment group. ANOVA results showing F-values (test statistic indicating variance among groups), and P-values (statistical significance). The lower section presents Tukey's Honest Significant Difference (HSD) post-hoc test results, showing pairwise differences between Treatments, with corresponding confidence intervals and P-values.

Shannon Diversity				
	CONTROL	3NOP	OIL	3NOP+OIL
**Shannon index values**	3.03	3.37	2.75	2.83
**ANOVA Test**				
	Df	F-Value	P-Value	
**Treatment**	3	277.33	<0.01	
**Period**	3	13.67	<0.01	
**Tukey's Honest post-hoc test - Treatment**				
	Difference	Lower	Upper	P-value
**3NOP vs. OIL+3NOP**	-0.59	-0.68	-0.51	<0.01
**CONTROL vs. 3NOP**	0.00	-0.08	0.07	1.00
**CONTROL vs. 3NOP+OIL**	0.59	0.51	0.67	<0.01
**OIL vs. 3NOP+OIL**	-0.07	-0.16	0.02	0.16
**OIL vs. CONTROL**	-0.66	-0.74	-0.58	<0.01

Table S2: PERMANOVA results for AF community composition showing R^2 values (proportion of variance explained), F-values (test statistic indicating group separation), and P-values (statistical significance). The lower section presents pairwise β-dispersion (homogeneity of variance) test P-values for Treatment groups.

PERMANOVA Test				
	Df	R^2	F-value	P-value
**Treatment**	3	0.36	53.12	<0.01
**Period**	3	0.10	14.35	<0.01
**Type**	1	0.02	7.58	<0.01
**Residual**	99	0.52		
**Homogeneity of Variance Test - Treatment**				
	**3NOP**	**3NOP+OIL**	**CONTROL**	**OIL**
**3NOP**	1.00	<0.01	0.42	<0.01
**3NOP+OIL**	<0.01	1.00	<0.01	0.63
**CONTROL**	0.41	<0.01	1.00	<0.01

OIL	<0.01	0.61	<0.01	1.00

Table S3: PERMANOVA results for AF community composition, comparingly only the CONTROL-fed samples in each period (P1-CON, P2-CON, P3-CON, P4-CON), showing R^2 values (proportion of variance explained), F-values (test statistic indicating group separation), and P-values (statistical significance). The lower section presents pairwise PERMANOVA results for each individual comparison

PERMANOVA				
	Df	R^2	F-value	P-value
**Group**	1	0.10	8.54	<0.01
**Period**	3	0.41	11.82	<0.01
**Type**	1	0.01	1.02	0.38
**Pairwise comparisons - Period**				
	R^2	F-value	PERMANOVA P-value	Procrustes P-value
**P1-CON vs. P2-CON**	0.27	7.85	<0.01	0.52
**P1-CON vs. P3-CON**	0.38	13.27	<0.01	0.13
**P1-CON vs. P4-CON**	0.27	8.07	<0.01	0.43
**P2-CON vs. P3-CON**	0.51	21.79	<0.01	NA
**P2-CON vs. P4-CON**	0.13	3.00	0.04	NA
**P3-CON vs. P4-CON**	0.37	13.19	<0.01	NA

Table S4: Real-time PCR results. Summary of linear mixed-effects model results, showing the effect estimates, standard errors, degrees of freedom (Df), T-values (test statistic for group differences), and P-values (statistical significance) for Treatment comparisons. The lower section presents Tukey's Honest Significant Difference (HSD) post-hoc test results, showing pairwise contrasts between treatments with corresponding estimates, T-values, and P-values.

**Linear Mixed Effects Model**					
**Effect**	**Estimate**	**Std. Error**	**Df**	**T-value**	**P-value**
Treatment 3-NOP	1.09	1.43	179.00	0.77	0.45
Treatment 3-NOP+OIL	-12.42	1.43	179.00	-8.69	<0.01
Treatment OIL	-12.99	1.43	179.00	-9.09	<0.01
**Tukey's Honest Post-hoc Test - Treatment**					
**Contrast**	**Estimate**			**T-ratio**	**P-value**
CONTROL vs. 3NOP	0.39	-	-	0.66	0.91
CONTROL vs. 3NOP+OIL	6.72	-	-	11.52	<0.01
CONTROL vs. OIL	5.79	-	-	9.92	<0.01
3NOP vs. 3NOP+OIL	6.34	-	-	10.86	<0.01
3NOP vs. OIL	5.40	-	-	9.26	<0.01
3NOP+OIL vs. OIL	-0.94	-	-	-1.60	0.38
